# Explainability Metrics of Deep Convolutional Networks for Photoplethysmography Quality Assessment

**DOI:** 10.1109/access.2021.3054613

**Published:** 2021-01-26

**Authors:** OLIVER ZHANG, CHENG DING, TANIA PEREIRA, RAN XIAO, KAIS GADHOUMI, KARL MEISEL, RANDALL J. LEE, YIRAN CHEN, XIAO HU

**Affiliations:** 1 Department of Computer Science, Stanford University, Stanford, CA 94305, USA; 2 Department of Electrical and Computer Engineering, Duke University, Durham, NC 27708, USA; 3 Institute for Systems and Computer Engineering, Technology and Science, 4200-465 Porto, Portugal; 4 School of Nursing, Duke University, Durham, NC 27708, USA; 5 Department of Neurology School of Medicine, University of California at San Francisco, San Francisco, CA 94110, USA; 6 School of Medicine, University of California at San Francisco, San Francisco, CA 94110, USA; 7 Department of Biostatistics and Bioinformatics, School of Medicine, Duke University, Durham, NC 27708, USA; 8 Department of Surgery, School of Medicine, Duke University, Durham, NC 27708, USA

**Keywords:** Deep neural network, PPG signal quality, biomedical informatics

## Abstract

Photoplethysmography (PPG) is a noninvasive way to monitor various aspects of the circulatory system, and is becoming more and more widespread in biomedical processing. Recently, deep learning methods for analyzing PPG have also become prevalent, achieving state of the art results on heart rate estimation, atrial fibrillation detection, and motion artifact identification. Consequently, a need for interpretable deep learning has arisen within the field of biomedical signal processing. In this paper, we pioneer novel explanatory metrics which leverage domain-expert knowledge to validate a deep learning model. We visualize model attention over a whole testset using saliency methods and compare it to human expert annotations. Congruence, our first metric, measures the proportion of model attention within expert-annotated regions. Our second metric, Annotation Classification, measures how much of the expert annotations our deep learning model pays attention to. Finally, we apply our metrics to compare between a signal based model and an image based model for PPG signal quality classification. Both models are deep convolutional networks based on the ResNet architectures. We show that our signal-based one dimensional model acts in a more explainable manner than our image based model; on average 50.78% of the one dimensional model’s attention are within expert annotations, whereas 36.03% of the two dimensional model’s attention are within expert annotations. Similarly, when thresholding the one dimensional model attention, one can more accurately predict if each pixel of the PPG is annotated as artifactual by an expert. Through this testcase, we demonstrate how our metrics can provide a quantitative and dataset-wide analysis of how explainable the model is.

## I. INTRODUCTION

Photoplethysmography (PPG) utilizes optoelectronic technology to detect changes in the blood volume of tissues, from which it can infer physiological signals and characteristics of the human body. It is used to monitor cardiac function, detect arrhythmia, and measure the blood-oxygen saturation level, heart rate, and heart rate variability [1]. Conventionally, this analysis has been done by traditional machine learning approaches [2]–[4]. However, in more recent years, deep learning models for PPG signal are becoming more and more prevalent [5]–[9]. With this shift in methodology in both processing PPG and doing artifact detection, a need for the interpretability of these deep learning models has arisen. Because of their complexity, deep learning models have become so-called “black boxes,” often achieving great performance without revealing how these decisions are being made. Moreover, deep learning models do not always act in a reasonable manner; they can suffer from problems like biased training sets [10] or simply poor decision-making logic [11]. In high stakes situations like healthcare one erroneous algorithm, without human oversight, could lead to terrible outcomes. Therefore, to catch these mistakes as well as to enable healthcare practitioners to more widely adopt these deep learning models, more and more research is being done into understanding the decision making process of these deep learning models [12]–[14]. Finally, explaining these deep learning models is especially important in PPG quality assessment. There are a set of tasks, such as artifact localization, estimating signal and artifact proportions, or artifact segmentation, which may be solved using deep learning models’ explanations. In sum, explainable models for PPG quality assessment have robust performance and can be useful in solving auxiliary tasks.

Currently, in the literature, there exists a variety of different methods to understand deep learning algorithms, some of which can be applied to signal processing and some of which cannot. One popular class of methods for visualizing neural network attention are saliency based methods such as Deconvolutional Saliency [15], Guided Saliency [16], and Integrated Gradients [17], among others [18], [19]. Such methods produce a heatmap quantifying the importance of different parts of the image or signal. However, these methods are local, only explaining the behavior of a deep learning model on a single image. They are also qualitative and difficult to use in rigorous comparison of different deep learning models. There exist other techniques, like Decision Tree Regularization or Activation Maximization, which do explain the model’s global behavior [18], [20]–[22]. Yet, each of these techniques are not readily applicable to models for processing signals. Decision Tree Regularization has difficulty dealing with high dimensional signal or image data, whereas Activation Maximization is qualitative and does not provide a rigorous way to compare models.

In this paper, our contributions are as follows. First, we provide novel explainability metrics which leverage domain-based expert knowledge to validate the reasoning behind a given deep learning model’s predictions. Our approach compares the neural networks’ attention to a human expert’s annotations and measures the difference over the whole dataset. Our first metric, *Congruence*, calculates the proportion of model attention within human annotations. Our second metric, *Annotation Classification*, thresholds the model attention to determine whether model attention covers all of the human annotations. The key difference between metrics is that Annotation Classification considers the spread of model attention, where Congruence does not. These metrics are general and can be easily applied to other signals provided the proper expert annotations. Second, we demonstrate using these metrics to perform comparative analysis. We train a new one-dimensional convolutional network on PPG artifact detection and use the explainability metrics to compare it to our previous model. Since the metrics are inherently numerical and are aggregated over the whole dataset, they provide a framework for the objective, global comparison of the explainability of different models. Finally, we perform experiments on our explainability metrics and find weak to moderate correlations (correlation coefficient around 0.3–0.4) between accuracy and explainability, while none of these correlations reach statistical significance after Bonferroni correction for multiple corrections. This reinforces the idea that more explainable models tend to be more reasonable and therefore accurate, but it also provides evidence that explainability cannot be quantified through accuracy alone. These explainability metrics, thus, quantify a different dimension of the model, beyond its performance.

In upcoming sections of the paper, we will adhere to the following terminologies. A model is considered “explainable” if it can consistently provide justifications or “explanations” for its decisions which agree with expert analysis. In this paper, our models are deep convolutional neural networks which give explanations in the form of attention maps. Additionally, a model is considered “reasonable” if it actually makes decisions in a similar fashion to experts. The saliency-based attention methods we use have strong theoretic reasons why the attention maps they produce mirror the model’s actual decision-making process [17], [19]. Thus, in our context, “explainable” models have a high chance of being “reasonable.”

## II. RELATED WORK

### A. PHOTOPLETHYSMOGRAPHY SIGNAL PROCESSING

We first review the relevance of deep learning in PPG signal processing before looking into the approaches to explaining convolutional neural networks. Deep learning has become more and more widespread in analysis of PPG signal. CorNET and Deep PPG are two popular neural network architectures for heart rate estimation based on PPG, which use the end to end deep neural network approach to outperform the traditional machine learning methods [5], [8]. PPG-based atrial fibrillation detection is another widely developed area which heavily uses deep neural networks [7]. For example, Kwon *et al.* compared the performance between 1D-CNN and RNN on 75 patients, concluding that 1D-CNN has the best performance [6]. Also, Shen *et al.* achieved 95% AUC on the 0.4M records dataset annotated by clinicians, and the 10K records from the NSR dataset [9]. These studies tend to be done in ambulatory settings, creating the most similar conditions to the final application. Additionally, these works lean to use large datasets in order to cover all the variability of the population. Based on this, promising results have been achieved using deep learning models to detect AF from PPG signal in outpatients [9], [23]–[25]. With this rise in deep learning in healthcare, as Ahmad *et al.* notes, there has been a recent push towards explainable or interpretable Machine Learning [12]. Black box models are powerful tools for medical purposes, yet must be treated with caution [13], [14]. They are susceptible to many different issues from data leakage to unrepresentative datasets, which render the models ungeneralizable.

### B. DEEP LEARNING INTERPRETABILITY

In recent years, a lot of work has been done with model interpretability. Interpretability techniques for machine learning can be broken up into two classes: local explanations and global explanations [12], [13]. Essentially, local explanations explain the model behavior on individual training instances, whereas global explanations explain the model behavior over the whole dataset. We first discuss the applications of various global methods to interpreting signal processing models before surveying the local explanatory methods.

#### 1) GLOBAL EXPLANATORY METHODS

Activation Maximization is one class of explanatory methods which has recently gathered an increased amount of attention due to its ability to generate human-interpretable representations of different filter within a convolutional network. The first paper to apply Activation Maximization to convolutional networks was Simonyan *et al.*, who used backpropagation to modify the input image to maximize the activation of an intermediate convolutional filter [18]. Subsequent papers have used techniques like center-biased regularization or generative modeling to improve the human interpretability of the images generated [20], [21]. Such an approach, however, might not be fit for explaining signal-based methods. First, signal processing is less intuitive than image recognition, so the images produced by Activation Maximization may be less understandable in the field of signal processing. Second, Activation Maximization is unable to do rigorous and quantitative analysis. Other global explanatory methods, including Tree Regularization or Network Dissection, cannot be applied to signal processing for other reasons [22], [26]. Tree Regularization seems unfit for general signal processing as decision trees become difficult to interpret in high dimensional data, and signal-processing lacks the highly annotated dataset which Network Dissection requires.

#### 2) LOCAL EXPLANATORY METHODS

We now direct our attention to local explanatory methods. One relevant class of locally explainable models is convolutional networks augmented with attention [27]. In such a case, the convolutional networks are trained to limit their vision to specific parts of the image, using network architecture which purposefully removes irrelevant information. From each image, then, it is possible to reclaim a map of the model’s attention, which would act as the explanation of the model’s behavior. To establish a fair comparison between our current model and our previous model, we did not adopt such an approach, as it would have required architectural changes. However, it should be noted that our method of comparing model attention and expert attention would work just as well on these attention maps.

Perhaps the most widespread class of local explanations are saliency methods. These methods use backpropagation or variants of backpropagation to track which areas of the image influence the model’s predictions the most. To our knowledge, the first such saliency method was deconvolutional saliency by Zeiler and Fergus, yet since then, many different methods have been developed based on this initial deconvolutional saliency [15]–[19]. In this paper, we use guided saliency, DeepSHAP, and integrated gradients. In brief, guided saliency involves taking the gradients of the model with respect to the input image but backpropagating through ReLU activations differently than deconvolutional saliency. Integrated gradients is done by taking the gradients on the interpolations between a reference image and the original image, and averaging these gradients. DeepLIFT, and later DeepSHAP, similarly uses a reference image, but instead of calculating derivatives, it calculates “difference-from-reference” values back throughout the image using a novel algorithm similar to backpropagation. These methods can identify model attention on a single image, but they are local to that single image. Such attention maps, then, may be unrepresentative of a wider trend. Similar to other methods, they are also not quantitative and therefore have trouble providing a rigorous approach to comparative analysis between models.

## III. METHODS

We designed a method for aggregating local attention maps to find global, quantitative explainability metrics which can be used for rigorous comparisons between deep convolutional networks. Essentially, we visualize a convolutional model’s attention via DeepSHAP, Integrated Gradients, or Guided Saliency and compare these maps to expert annotations. Our two metrics are *Congruence*, which measures the proportion of the model’s attention within the expert annotations (the validity of the model attention), and *Annotation Classification*, which roughly measures the proportion of expert annotations covered by the model’s attention (the spread of the model attention). We reason that if the model’s attention highlights regions which the annotators marked as essential, then the model’s attention can be considered explainable. Our explainability metrics were applied to two deep learning models on PPG Signal Quality Index Assessment. One was a two-dimensional ResNet18, which used He *et al.*’s original architecture, and the other was a specialized one-dimensional ResNet34, which used Dai et al’s proposed architecture [28], [29]. There were 78278 instances in the training set and 2683 instances in the testing set. Moreover, the onset and offset of artifact in each of the testing set segments were annotated, and these annotations were used to compute our explainability metrics. In the following subsections, we first describe the model architecture and training and testing procedure before examining the mathematics behind each explainability metric.

### A. DEEP LEARNING BASED PPG SQI ASSESSMENT

#### 1) ResNet ARCHITECTURE

ResNet, short for Residual Network, is a classic neural network architecture. It first appeared in 2015, winning that year’s Image Net competition, and has since become widespread in a variety of different computer vision tasks [29]. The fundamental breakthrough of ResNet stemmed from the concept of the “residual block.” Each block stacks convolution layers as before, yet also adds the original input to the output of the convolution block. This “skip-connection” mitigates the problem of vanishing gradients, allowing deeper models to be trained successfully. In our study, we use Dai *et al.*’s Resnet-34 for 1D signal data and the vanilla Resnet-18 for 2D image data [28], [29]. The architecture and weights of the two dimensional Resnet-18 comes from our previous paper [30].

### B. TRAINING AND VALIDATION PROCEDURE

In terms of our hardware setting, the training process of the model was run on a machine with Intel I9–7900K with 128GB RAM. Besides that, one Nvidia GTX 2080Ti was used for tensor computation. Inside of the model, GLOROT_UNIFORM is selected as the initializer function for each convolution kernel with the kernel size of 80. During training, we applied binary cross entropy as the loss function and the Adam optimizer with 1e-4 as the learning rate [31]. The models were trained for fifty epochs, and after each epoch, the performance on the validation set was calculated. In the end, the model at the end of the epoch with the best performance on the validation set was selected as the final model.

### C. NOTATION FOR EXPLAINABILITY METRICS

We visualized the model’s attention over the testing dataset consisting of 825 artifactual examples. Each example is denoted (*x*_*i*_, *z*_*i*_). Here, *x*_*i*_ is a 30-second one dimensional PPG signal broken up into 7201 separate data points *x*_*i,j*_. Each *z*_*i*_ represents the human annotations and can also be broken down into 7201 data points *z*_*i,j*_. We have that *z*_*i,j*_ is 1 if the datapoint is within the human annotations, 0 if not. Finally, the model’s attention on *x*_*i*_ is denoted ${\hat{z}}_{i}$ and is also indexed by *j*. This model attention is acquired by using one of DeepSHAP, Guided Saliency, or Integrated Gradients, and then taking the absolute value of these measurements, as discussed in section III.D. Note that for the image-based ResNet, the model’s attention will be two dimensional. To calculate the one dimensional model attention vector ${\hat{z}}_{i}$, we take the maximum attention over the columns.

### D. CALCULATING MODEL ATTENTION ON A SINGLE PPG SEGMENT

**FIGURE 1.**
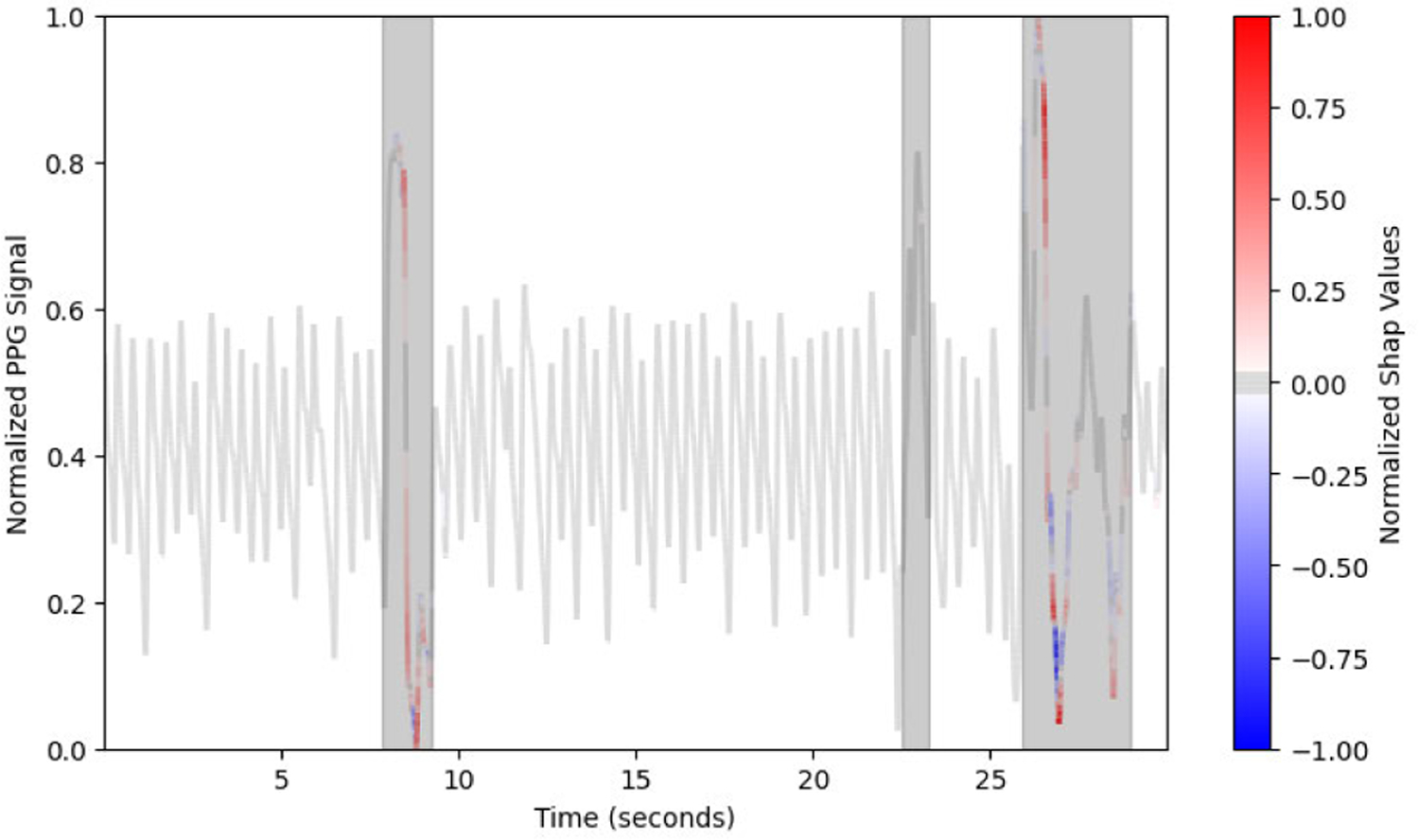
When using DeepSHAP to visualize model attention, the model ignores all non-artifactual PPG data, and instead focuses on the artifactual PPG sections when assigning both positive and negative contributions for the training example being artifactual. Here, shaded sections are artifactual as annotated by experts.

To calculate the attention of a convolutional neural network on a single PPG segment, one should apply one of DeepSHAP, Guided Saliency, or Integrated Gradients. The absolute value of these saliency maps can be considered the model’s attention. This is because, as shown in Figure 1, both positive and negative contributions are usually within the human annotated regions. If any part of the example contains artifact, then the whole example is deemed artifactual, so an optimal predictor would ignore non-artifactual PPG. Instead, the model must pay careful attention to the most irregular PPG sections and determine if the irregular PPG is artifactual or not. Therefore, we hypothesize that the reasons deterring the model from classifying the PPG as artifactual will exist in the irregular sections. The absolute values of DeepSHAP or Integrated Gradients better quantify the model’s attention. Note that we need not take the absolute values of Guided Saliency because Guided Saliency already removes all negative contributions.

### E. CONGRUENCE

Congruence, our first explainability metric, measures the proportion of the model attention which is within the human annotations. It measures whether the model is looking at a reasonable place when making decisions and is defined on a single example as follows:

(1) $$\text{Cong}\left(z_{i},{\hat{z}}_{i}\right)=\frac{z_{i}\cdot {\hat{z}}_{i}}{\sum_{j}{\hat{z}}_{i,j}}=\frac{\sum_{j}z_{i,j}\cdot {\hat{z}}_{i,j}}{\sum_{j}{\hat{z}}_{i,j}}$$

Note that *z*_*i,j*_, the human annotation, has value either zero or one. The denominator measures the total amount of model attention; the numerator measures the total amount of model attention with the human annotations. Congruence then measures the proportion of the model attention which is within the human annotations. To calculate congruence over a whole dataset, the we average the congruences of each datapoint in that dataset.

### F. ANNOTATION CLASSIFICATION

Importantly, congruence does not provide information about the coverage of the model’s attention. Congruence may be equal to one, even if all of the model’s attention is focused on only one pixel within the human annotations. To measure the coverage of the model’s attention on the human annotations, we propose a second explainability metric, annotation classification. This metric measures the difference in the attention maps between the annotated and nonannotated regions of the signal. Each PPG segment is broken down into multiple smaller segments and the maximum attention on each segment is thresholded to predict or “classify” whether the data was annotated as essential or nonessential. The threshold starts at zero and iterates, in sorted order, through each segment’s maximum attention value. Consequentially, every change in the segment classification is recorded. At each given threshold, the number of true positives and false positives for this essential/nonessential classification task can be calculated. By iterating through thresholds in monotonically increasing order, a full ROC curve can be constructed. The ROC curve here represents how much predictive power the model attention has in predicting the importance of the smaller data segment. Finally, the AUROC is our metric and a quantitative measure of the ROC. Variations of this metric can be derived from this method and differ in how each datapoint is broken down into smaller segments.

#### 1) PIXEL ANNOTATION CLASSIFICATION

The first metric is named “Pixel Annotation Classification” and involves breaking down each data point into pixels. For each pixel, we threshold ${\hat{z}}_{i,j}$ to classify whether each pixel is in the expert annotated zones. Finally, we take the AUROC to be our metric. This gives us a measure of how similar model attention is to human annotations on a pixel level.

#### 2) SECTIONAL ANNOTATION CLASSIFICATION

The second classification metric is named “Sectional Annotation Classification.” Instead of thresholding the pixel values directly, we break each example into different sections, as marked out by the human annotations. Then we consider the maximum model attention within each section and threshold it to classify whether the section is annotated as artifactual or non-artifactual. Because artifactual and non-artifactual have different sizes on average, it is possible taking the maximum model attention within each section introduces some bias. We further discuss the potential impact of this bias in the limitations subsection of the discussion (section VI.F).

#### 3) INTERVAL ANNOTATION CLASSIFICATION

The final metric is named “Interval Annotation Classification.” We now take the maximum model attention on fixed intervals which remain constant over all the images. For instance, we broke our 30 second PPG segments into six 5-second intervals. If any portion of the interval was deemed artifactual by the human annotations, the whole interval was counted as artifactual. Then we thresholded the maximum model attention over these fixed intervals to do classification.

## IV. COHORT

### A. COHORT SELECTION

#### 1) TRAINING SET

Biomedical signals were acquired from 3764 patients in the intensive care unit (ICU) from 3/2013 to 12/2016 [32]. The training set was composed of four 30-sec segments of signals which were randomly selected and extracted from each patients’ bedside monitors. The retrospective use of this automatically archived dataset for this study was approved by UCSF IRB with a waiver of patient consent (IRB approval number: 16–18764).

#### 2) TEST SET

The test set comes directly from our previous work [30] where more details can be found. It consists of 13 stroke patients who had entered the Neurointensive Care Unit at UCSF from October 2016 to January 2018 [30]. To be added to the test set, each patient need to be diagnosed with acute ischemic stroke and give proper consent. The guidelines for proper consent was that each patient was at least 18 years old and capable of understanding the protocols to which they were consenting. For instance, all patients spoke English and those with significant problems related to their attention, alertness, cognitive function, or communication were excluded unless a legally authorized representative could consent on their behalf. All enrolled patients provided written consent after being informed of the protocols approved by UCSF’s Institutional Review Board.

In total, between three and twenty-two hours of biomedical signals were recorded per stroke patient. Each patient’s data was further broken down into multiple 30-second segments. Eight of the thirteen patients had atrial fibrillation episodes already documented by the clinicians at the time of admission on the Neurointensive Care Unit.

### B. ANNOTATION

This section outlines the annotation process for the training and test set. The labeling and annotating was done and described in our previous work [33].

#### 1) QUALITY LABELING

A subset of randomly selected PPG segments from training set were assigned to the labelers without overlapping. For the test set, a subset of randomly selected PPG segments from the cohort of stroke patients was assigned to all labelers (*n* = 3). The test set consisted only of those segments that all labelers agree on, ensuring a congruent test set (2683 out of 3000 records). During the labeling process, the segments can be labeled as: Good Quality, Bad Quality or Not Sure. From our previous work, we created a definition for a good quality segment based on the physiological context, that need to fulfil the rules: 1) reflect the response of blood volume to the underlying pathophysiological characteristics of the cardiovascular system, irrespective of the particular shape of the pulse; 2) show a consistent number of inflection points; 3) be artifact-free and 4) be free of irregular shapes that cannot be explained by ECG changes [33].

#### 2) PERCENTAGE OF ARTIFACT

We annotated each PPG segment in the test set by marking the onsets and offsets of artifactual within a 30-second segment. These annotations were not used as class labels and did not affect the training process, but instead were used for validating the explainability of the deep learning model’s attention. As opposed to assigning labels, the annotation of artifact was done by a single person.

## V. RESULTS ON REAL DATA

### A. EVALUATION APPROACH

We first compared the different models’ performances on the testing set to see how well each model discerns artifactual from non-artifactual PPG. Then we evaluated each of the models’ different explainability to validate their performances. Finally, as a follow up study, we measured how correlated explainability and model performance is. We do not perform comparisons between our metrics and other explainability metrics because we were unable to find such metrics to do proper comparisons. This is further examined in the discussion section.

### B. MODEL PERFORMANCE ON THE TESTING SET

After training, performance on the testing set was determined. To binarize the classification output into discrete categories (artifactual or artifact-free), a threshold of 0.5 was used. Subsequently, sensitivity, specificity, and accuracy were calculated. The one dimensional ResNet model maintains a higher sensitivity, whereas the two dimensional ResNet model, presented in the previous paper, has a higher specificity [30]. Both two dimensional and one dimensional models have a good accuracy (*>* 98%), but the one dimensional ResNet model also outperforms the two dimensional model.

### C. MODEL EXPLAINABILITY ON THE TESTING SET

Table 2 shows the performance of our models when aggregating the attention maps generated by different explanatory metrics. For instance, “DeepSHAP” refers to how we generate the attention maps of each individual PPG segment, and “Pixel” refers to how we aggregate these maps. The terms Pixel, Sectional, and Interval represent Pixel-based, Sectional-based, and Interval-based Annotation Classification, respectively. For each metric, the one dimensional model has higher explainability than the two dimensional model, invariant of the explanatory metric. On average, the one dimensional model has 0.1475 higher Congruence and 0.0975 higher Annotation Classification score than the two dimensional ResNet. Additionally, both models seem to be best at Interval Annotation Classification, and worst at Pixel Annotation Classification. Over both models, Interval, Sectional, and Pixel Classification have average Annotation Classification scores of 0.761, 0.686, and 0.652, respectively.

### D. DETERMINING CORRELATION BETWEEN EXPLAINABILITY AND PERFORMANCE METRICS

**FIGURE 2.**
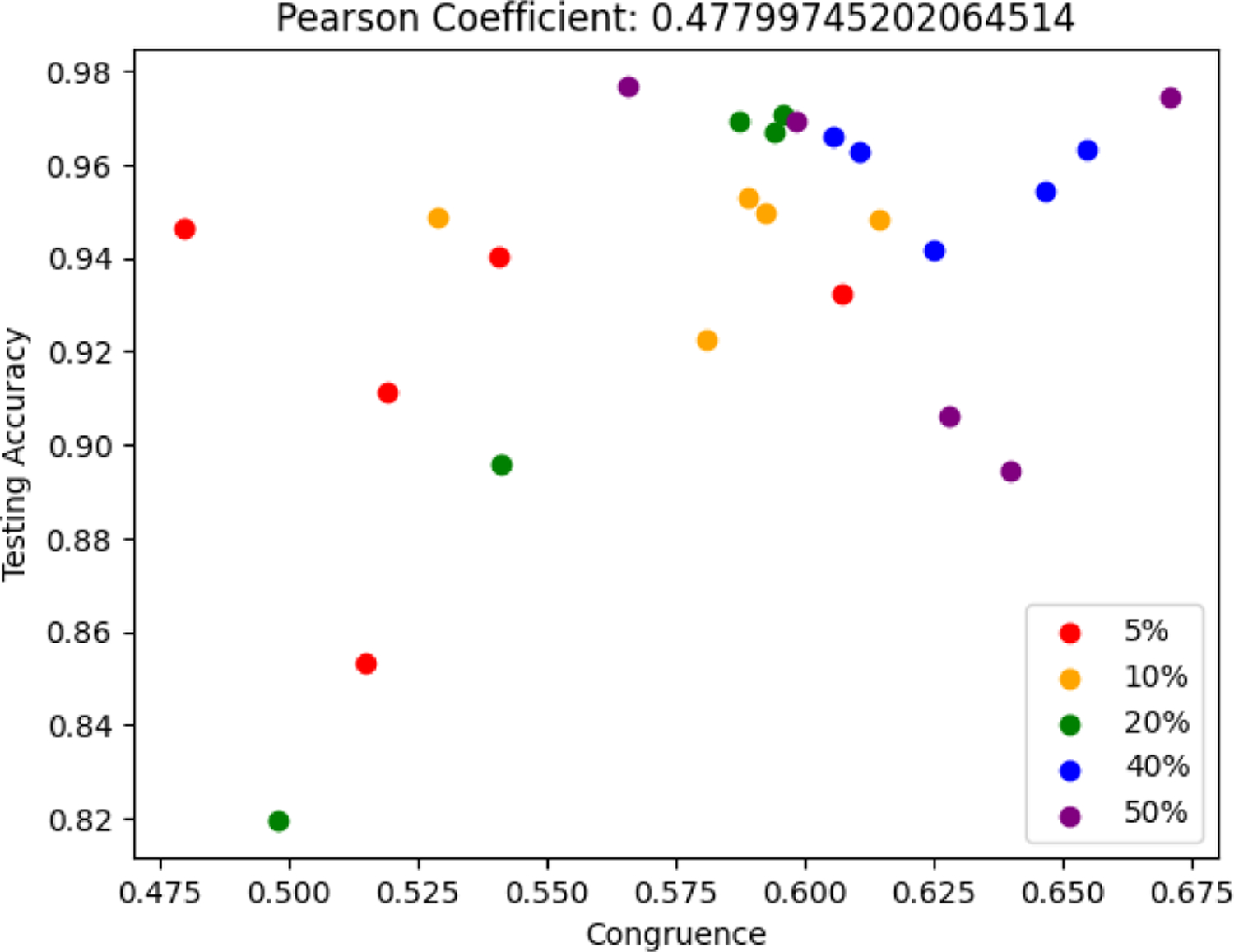
A scatterplot of the model’s Congruence against its Testing Accuracy. Models have been color coded depending on what proportion of the data they were trained on.

To determine the consistency of our different explanatory metrics with performance metrics, such as accuracy, NPV, and specificity, we ran a total of 25 additional one dimensional ResNet models with the same hyperparameters and architecture as before. For these 25 models, we varied the dataset size, using 5%, 10%, 20%, 40%, and 50% of the data to induce overfitting, so that the testing accuracy would be varied. Specifically, for each dataset size, we trained five separate models. Furthermore, as the explanatory metrics are only defined on artifactual data, and artifactual data is classified as ‘negative’ by the model, we considered NPV and Specificity without considering PPV and Sensitivity. We observed weak to moderate correlation between the different explanatory metrics and performance metrics, although most of them don’t reach statistical significance (*p >* 0.05) and none after adjustment for multiple comparisons (*p >* 0.008). Some models which were fairly accurate (*>*90%) performed poorly on the explanatory metrics, whereas other models which performed well on the explanatory metrics were relatively inaccurate in classifying the testing set. In general, however, more explainable models tended towards being more accurate. The Pearson correlation coefficients calculated on the entire set of models have been displayed in Table 3. Beyond that, Figure 2 contains a scatterplot comparing Congruence to Testing Accuracy. Here, the points have been colored to show how dataset size influences this correlation. For more details on how dataset size affects performance and explainability metrics, see Figure S1 and Table S1 in the supplementary materials.

## VI. DISCUSSION

### A. IMPORTANCE OF EXPLANABILITY METRICS

Explainability is important in any application which uses deep learning, especially in the healthcare domain. For high-stakes environments like healthcare, domain experts often require insight into the model’s decision making process. Our explainability metrics quantify whether a given deep learning model is explainable, or whether the explanations provided by visualizing the model’s attention align with expert annotations. This increases domain experts’ insight and therefore trust of a given deep learning model. Moreover, if one accepts that the explanations provided by the model are a good proxy for the model’s true decision-making process, then ensuring that a model is explainable also helps prevent unreasonable models from being deployed. Explainability metrics then enable quantitative comparison of different model’s reasonableness.

Explainability is especially important in PPG quality assessment for a variety of reasons. Beyond robustness and building trust, a model with explainable attention maps can perform tasks such as artifact localization, estimating signal and artifact proportions, or even artifact segmentation. In other words, the attention maps of a deep learning model trained to do PPG quality assessment have the potential to complex localization tasks. We plan to explore this in future works.

### B. DIFFERENCES BETWEEN CONGRUENCE AND ANNOTATION CLASSIFICATION

The key difference between Congruence and Annotation Classification is that Congruence does not consider the coverage of expert annotation, whereas Annotation Classification does. A model with high Congruence and low Annotation Classification may have most of its attention concentrated on a few pixels within expert annotation boundaries. Such a model may have high Congruence, as a large proportion of its attention is valid. However, when thresholding to do annotation classification, the model attention may only be used to classify a small proportion of the intervals or pixels properly. In this way, Annotation Classification rewards broad coverage of each artifactual section/interval/pixel.

### C. COMPARISON OF MODELS

The attention maps of the one dimensional model are more similar to the human annotations than the attention maps of the two dimensional model. This is corroborated by all the different combinations of metrics and saliency method; in all of these fields, the one dimensional model performs in a more explainable manner than the two dimensional model. This comparison helps demonstrate how simple it is to do model comparisons with these explainability metrics. With our quantitative analysis, one might even be able to design an algorithm similar to hyperparameter tuning which runs multiple models and optimizes for a combination of performance and explainability. Furthermore, this analysis is not limited to PPG data; all the steps are repeatable on other signals such as Electrocardiogram or Electroencephalogram signals. The only limiting factor is expert annotations of the sections of the signal important for signal analysis.

### D. WEAK TO MODERATE CORRELATION BETWEEN EXPLANATORY AND PERFORMANCE METRICS

To understand the weak to moderate correlation between the explanatory and performance metrics, we examine some non-intuitive models. Some models have high accuracy but low explainability. These models often fall into two classes. Either their attention is scattered uniformly on the example, or their attention is concentrated on the non-artifactual sections of the example. In both cases, the explainability metrics uncover the fact that deeper analysis is required on such models before the models are used in a real-world setting. The converse may happen as well; some models have high explainability but low accuracy. This can happen when the bias term for the model’s final feedforward layer is wrong; the model may predict ‘non-artifactual’ for each example regardless of the actual data. Surprisingly, some of these biased models actually pay attention to the artifactual data in making their predictions. We conclude that the model is learning valid pattern recognition, yet this is being out-weighed by the model’s bias term. In either case, the inconsistent and non-significant correlations between explanatory and performance metrics indicate additional insights can be offered by proposed metrics on top of conventional performance metrics about the deep learning models.

### E. LACK OF BASELINES

When introducing a new metric, it is recommended to compare the proposed metric to other baseline metrics. However, to our knowledge, existing metrics applicable to signal processing do not offer global and quantitative analysis of model attention, so a comparison with other metrics is difficult. For instance, a comparison between the proposed metrics and saliency methods is uninformative, as the proposed methods are global, whereas saliency methods are local. Our metrics are not designed to replace saliency methods, but instead aim to provide a system for rigorous comparisons of saliency attention maps. In other words, our metrics are explicitly based on saliency methods. Finally, both saliency methods and activation maximization are qualitative, so comparison with these metrics is difficult as well. Instead, the proposed metrics are valuable because they are the first globally representative, yet quantitative metrics which can be applied to signal processing.

### F. LIMITATIONS

From a biomedical perspective, our dataset may have some limitations. For one, the size of our testing dataset is modest, at around 2700 data points. This size may not be representative to all the heterogeneities of the populations. For instance, we did not include other arrhythmias besides atrial fibrillation. Another limitation may come from our expert annotations on the testing dataset of where the artifact is localized; there may be high variability between annotators’ thoughts on where the artifact is. As we only had a single annotator, our annotations may be inaccurate. Note that the inaccuracy due to the single-annotator is expected to be moderate and tolerable, as the annotator was very experienced and was involved in all annotation projects. Moreover, label annotations had good agreement (Kappa coefficient 0.83) due to clear rules of what counts as artifact [33].

Our machine learning methods may have some limitations as well. It is possible that saliency methods do not truly reflect model attention, or that the model behavior cannot be quantified in simple heatmaps. Additionally, Sectional or Interval Annotation Classification uses the maximum attention over a section or interval, which may bias longer segments to be marked as more important. In the case of interval annotation classification, both artifactual intervals and non-artifactual intervals have the same length, so this is not a problem. However, the same cannot be said of Sectional Annotation Classification. As artifactual sections are on average much smaller than non-artifactual sections, this biases the non-artifactual sections to be marked as more important. As a result, the Sectional Annotation Classification may underestimate model explainability and should be used with caution when large variation in intervals exists across different conditions to be classified. Finally, our methods only analyze model attention on artifactual PPG segments, as those have the proper expert annotations. It is possible that if one was able to analyze model attention on the non-artifactual PPG, one would reach a wider understanding of the model’s behavior.

## VII. CONCLUSION

In this work, we presented two metrics, Congruence and Annotation Classification, for quantifying the validity of model attention maps by comparing them to expert annotations. Congruence measures the proportion of model attention within the expert annotations, and Annotation Classification indirectly measures the amount of expert annotations covered by model attention. These explainability metrics are both quantitative and global, making them well suited for rigorous model comparisons. We demonstrated an application of our metrics on a novel model for PPG artifact detection, and such an application is easily transferable to processing other signals, provided one has the right expert annotations. Finally, through some other experiments, we determined that model performance on PPG artifact classification was weakly to moderately correlated with model explainability. This provides evidence that these new metrics provide novel insights into the model which are not covered by performance metrics.

## Supplementary Material

supp1-3054613

## Figures and Tables

**TABLE 1.** The performance of the 1D and 2D models is similar.

Testing Set	ResNet1d	ResNet2d
Sensitivity	0.9947	0.9791
Specificity	0.9769	0.9877
Accuracy	0.9892	0.9851

**TABLE 2.** The 1D Model is Consistently More Explainable.

Dimension	One Dimension				Two Dimension			
Metric Used	Pixel	Sect.	Int.	Cong.	Pixel	Sect.	Int.	Cong:
DeepSHAP	0.6526	0.7477	0.7934	0.4825	0.5820	0.6010	0.6601	0.3737
Integrated Grad.	0.6073	0.7136	0.7665	0.4393	0.5951	0.6420	0.6887	0.3852
Guided Saliency	0.8061	0.7607	0.8864	0.6017	0.6689	0.6477	0.7709	0.3220

**TABLE 3.** There is Weak to Moderate Correlation between Explainability and Performance Metrics.

Metric 1	Metric 2	Correlation	P-Value
Congruence	Testing Accuracy	0.478	0.016
Sectional Classification	Testing Accuracy	0.060	0.776
Interval Classification	Testing Accuracy	0.333	0.104
Pixel Classification	Testing Accuracy	0.460	0.021
Pixel Classification	NPV	0.130	0.536
Pixel Classification	Specificity	0.103	0.626
