# Supplementary material for: Explainability Metrics of Deep Convolutional Networks for Photoplethysmography Quality Assessment
Source: IEEE Access. Author manuscript; Available in PMC 2021 Mar 19. (PMC7978398; doi:10.1109/access.2021.3054613)
Supplement: supp1-3054613 [file NIHMS1676349-supplement-supp1-3054613.pdf]

Supplementary Material

Here are the supplementary materials associated with ”Explainability Metrics of Deep Convolutional Networks for Photoplethysmography Quality Assessment.”

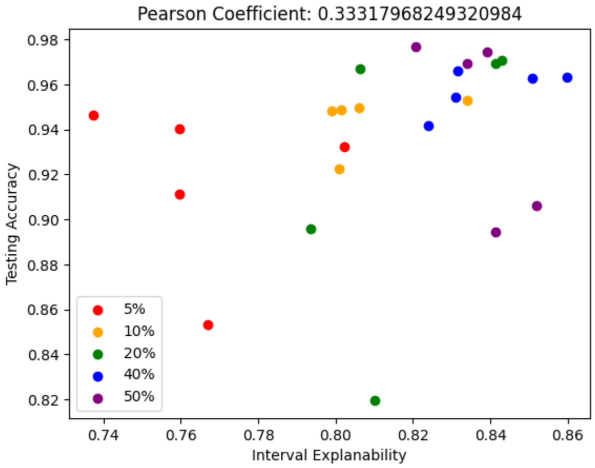

Figure S1: A scatterplot of the model's Interval Annotation Classification against its Testing Accuracy. Models have been color coded depending on what proportion of the data they were trained on.

Table S1: Training Dataset Proportion compared against Average Performance and Explainability Metrics

Proportion	Testing Accuracy	Congruence	Pixel	Sectional	Interval
5%	0.917	0.532	0.618	0.755	0.765
10%	0.944	0.581	0.666	0.765	0.808
20%	0.924	0.563	0.673	0.775	0.819
40%	0.958	0.629	0.716	0.769	0.839
50%	0.944	0.621	0.706	0.774	0.837
